# Supplementary material for: Restored Intensities from Customized Crops of NMR Experiments (RICC-NMR) to Gain Better Insight on Chemometrics of Sicilian and Sardinian Extra Virgin Olive Oils
Source: Foods. 2025 May 19;14(10):1807. doi: 10.3390/foods14101807 (PMC12110953; doi:10.3390/foods14101807)
Supplement: Supplementary file 1 [file foods-14-01807-s001.zip › foods-3622253-supplementary.pdf]

foods

Supplementary Material for the paper titled

Restored Intensities from Customized Crops of NMR experiments (RICC-NMR) to gain a better insight on chemometrics of Sicilian and Sardinian Extra Virgin Olive Oils.

Nicola Culeddu [1], Archimede Rotondo [2,*], Federico Nastasi [3], Giovanni Bartolomeo [2], Pierfrancesco Deiana [4], Mario Santona [4], Petros A. Tarantilis [5], Giuseppe Pellicane [2,6] and Giovanna Loredana La Torre [2,*]

1. CNR—Istituto di Chimica Biomolecolare (ICB), Traversa La Crucca 3, Loc. Baldinca, Li Punti, 07040 Sassari, Italy; nicola.culeddu@cnr.it
2. Department of Biomedical and Dental Sciences and Morpho-Functional Imaging (BIOMORF), University of Messina, 98168 Messina, Italy; gbartolomeo@unime.it (G.B.); gpellicane@unime.it (G.P.)
3. Dipartimento di Scienze Chimiche, Biologiche, Farmaceutiche ed Ambientali (CHIBIOFARAM), Università di Messina, 98166 Messina, Italy; federico.nastasi@studenti.unime.it
4. Dipartimento di Agraria, Università degli Studi di Sassari, 07100 Sassari, Italy; pideiana@uniss.it (P.D.); msantona@uniss.it (M.S.)
5. Laboratory of Chemistry, Department of Science, Agricultural University of Athens, 75 Iera Odos, 118 55 Athens, Greece; ptara@aua.gr
6. School of Chemistry & Physics, University of KwaZulu-Natal, Pietermaritzburg, Private Bag X01, Scottsville 3209, South Africa

* Correspondence: arotondo@unime.it (A.R.); llatorre@unime.it (G.L.L.T.); Tel.: +39-090-6765731 (A.R.); +39-090-6766315 (G.L.L.T.)

Academic Editor: Charis M. Galanakis

Received: 17 April 2025
Revised: 11 May 2025
Accepted: 15 May 2025
Published: 19 May 2025

Citation: Culeddu, N.; Rotondo, A.; Nastasi, F.; Bartolomeo, G.; Deiana, P.; Santona, M.; Tarantilis, P.A.; Pellicane, G.; La Torre, G.L. Restored Intensities from Customized Crops of NMR Experiments (RICC-NMR) to Gain Better Insight on Chemometrics of Sicilian and Sardinian Extra Virgin Olive Oils. *Foods* **2025**, *14*, 1807. https://doi.org/10.3390/foods14101807

Copyright: © 2025 by the authors. Licensee MDPI, Basel, Switzerland. This article is an open access article distributed under the terms and conditions of the Creative Commons Attribution (CC BY) license (https://creativecommons.org/licenses/by/4.0/).

Abstract: Herein we leave the most important data to recover the experiments and the material which allowed us to write the related article.

Keywords: NMR analysis; RICC-NMR, extra-virgin-olive-oil; *Olea europaea*; ^{1}H{^{13}C}-NMR, metabolic profile. PLS-DA

S1. Samples

As a case study to evaluate the application of our original procedure, described in Sections 2.3 and 2.4, we chose to compare two groups of extra virgin olive oils (EVOOs) from Sardinia and Sicily, for which the literature provides genetic evidence indicating a certain degree of genetic correspondence [19]. The thirty-seven chosen EVOO samples were selected from Sicilian (eighteen samples of *Nocellara* cultivar) and Sardinian (nineteen samples of *Bosana* cultivar) crops to provide a rationale explaining the *Olea Europaea* terroir condition. Specific goods were given by local producers in dark, sealed bottles after the 2023-2024 campaign. Samples were kept sealed in dark and dry cupboards until January 2025 and then picked up for analysis. Measurements were run in triplicate. After dilution in $CDCl_3$, sample solutions were analysed or kept in the -44°C freezer. According to the NMR profile, the frozen solution remains stable for 20 days.

S2. Details about Materials and Methods

S2.1 Matlab processing

As specified in the main text the analysis take the advantage to process data from three different datasets coming from ^{1}H NMR experiments (RICC-NMR) and then these are combined with other data coming from the ^{13}C-NMR to give a targeted output. It means that all data are translated into the specific quantitative contributions of given components.

Herein we report the code used together with the known "icoshift" routine in MATLAB to obtain datasets (integrations) later converted into quantification indicators through the MARA-NMR algorithm.

For [1]H.

```
############################################################
%% [1Hd]Import 1Hd spectra, organize those in tables
sp1Hdec = table2array(experimenttable1Hd(:,1:43));
% spec1Hdec = str2double(sp1Hdec);
a1Hd= round(mean(find(abs(sp1Hdec(:,1)+0.5)<0.0002)));
b1Hd= round(mean(find(abs(sp1Hdec(:,1)-10.5)<0.0002)));
F1Hdimport = sp1Hdec([a1Hd:b1Hd],[2:43]);
%% [1Hd]Normalize spectra and show the level AUC
X1Hdorigpt = 1:size(F1Hdimport,1);
% spectra running along columns and data-points transponed as well
F1Hdn = msnorm(X1Hdorigpt.',F1Hdimport,'Limits',[15000 15800],'Max',1000)
plot(F1Hdn);
%% [1Hd]Store 1Hd spectra, spectral witdh, data-points and resample with reference
ns=size(F1Hdn,2);
X1Hd = cell(ns,1);
Y1Hd = cell(ns,1);
T1Hd = cell(ns,1);
X1Hdn = cell(ns,1);
Y1Hdn = cell(ns,1);
rmi1Hd = zeros(ns,1);
rmx1Hd = zeros(ns,1);
shift = zeros(ns,1);
sw1 = 110000;
for i = 1: ns
    Y1Hd{i} = F1Hdn(:,i)
    % stse = strfind(Y{i}',[0 0 0])
    %torem = unique([stse, stse+1, stse+2])
    % Y1Hd{i}(torem,:) = []
    % Y1Hd{i} = flip(Y{i})
    T1Hd{i}= linspace(1,numel(Y1Hd{i}),numel(Y1Hd{i}))
    rmi1Hd(i) = round(0.104*numel(Y1Hd{i}))
    rmx1Hd(i) = round(0.11*numel(Y1Hd{i}))
    f = fit(T1Hd{i}(rmi1Hd(i):rmx1Hd(i)).', Y1Hd{i}(rmi1Hd(i):rmx1Hd(i)),'gauss1')
    shift(i) = f.b1*(sw1/numel(Y1Hd{i}))
    low1Hd(i) = 6833-shift(i)
    hi1Hd(i) = low1Hd(i)+sw1
    X1Hd{i} = linspace (low1Hd(i), hi1Hd(i), numel(Y1Hd{i}))
    X1Hd{i} = X1Hd{i}.'
```

```
[X1Hdn{i}, Y1Hdn{i}] = msresample(X1Hd{i},Y1Hd{i}, 100000,'RANGE',[0 100000],'Uniform', true,'showplot',true)
end
% delete the old Y1Hdsp every time
figure;
Y1Hdsp = transpose(cell2mat(Y1Hdn.'));
plot(Y1Hdsp.');
%% [1Hd]Show the NMR spectra as they are represented
% Yr = flip(Ysp.',1)
X1Hda = linspace (0, 10, 100000);
plot (X1Hda, Y1Hdsp.');
xlabel('shift(ppm)');
ylabel('Intensity');
set(gca,'Xdir','reverse');
%% [1Hd]Base-line correction, plot one correction and the stackplot
sf = 900;% best 700
pf =0.0003;% best 0.024
ws= 1500;% best 1600
wf = @(mz) sf+ pf .*mz;
%define smothing factor 'sf' and pace factor pf
X1Hdpt = 1:100000
Y1Hdblc = msbackadj(X1Hdpt.',Y1Hdsp.','StepSize', wf,'WindowSize',ws,'RegressionMethod','spline','ShowPlot',3);
% define ws =2600
%Yblcr = flip(Yblc,1)
figure;
plot(X1Hdpt,Y1Hdblc);
ylim([-100 300]);
xlabel('shift(ppm)');
ylabel('Intensity');
set(gca,'Xdir','reverse');
minval =min(Y1Hdblc);
meanmin= mean(minval);
disp(minval);
disp(meanmin);
%% [1Hd]Subtract the baseline height (delete previous BL1Hd and base1Hd before you run it)
Y1Hdbc = Y1Hdblc.'
BL1Hd = Y1Hdbc(:, [700:1400 4000:4700 33400:34000 44500:45300 47500:48700 57819:59228 73600:79370 84360:90000 97490:99000]);
base1Hd = mean(BL1Hd,2);
Y01Hd = Y1Hdbc-base1Hd;
plot(Y01Hd.');
%% [1Hmsd] Import multiple suppressed and 13Cdecoupled 1Hmsd spectra
sp1Hmsd = table2array(exptable1Hmsd(:,1:43));
% spec1Hdec = str2double(sp1Hdec);
a1Hmsd= round(mean(find(abs(sp1Hmsd(:,1)+0.5)<0.0005)));
b1Hmsd= round(mean(find(abs(sp1Hmsd(:,1)-10.5)<0.0005)));
F1Hmsdimp = sp1Hmsd([a1Hmsd:b1Hmsd],[2:43]);
%% [1Hmsd] Normalize 1Hmsd spectra and show the level AUC
X1Hmsd_orpt = 1:size(F1Hmsdimp,1);
```

```
% spectra running along columns and data-points transponed as well
F1Hsdn = msnorm(X1Hmsd_orpt.',F1Hmsdimp,'Limits',[17500 18500],'Max',1000)
plot(F1Hsdn);
%% [1Hmsd] Store spectra, spectral witdh, data-points and resample with reference
ns=size(F1Hsdn,2);
X1Hsd = cell(ns,1);
Y1Hsd = cell(ns,1);
T1Hsd = cell(ns,1);
X1Hsdn = cell(ns,1);
Y1Hsdn = cell(ns,1);
rmi1Hsd = zeros(ns,1);
rmx1Hsd = zeros(ns,1);
shift1Hsd = zeros(ns,1);
sw1H = 110000;
for i = 1: ns
    Y1Hsd{i} = F1Hsdn(:,i)
    % stse = strfind(Y{i}',[0 0 0])
    %torem = unique([stse, stse+1, stse+2])
    % Y1Hd{i}(torem,:) = []
    % Y1Hd{i} = flip(Y{i})
    T1Hsd{i}= linspace(1,numel(Y1Hsd{i}),numel(Y1Hsd{i}))
    rmi1Hsd(i) = round(0.104*numel(Y1Hsd{i}))
    rmx1Hsd(i) = round(0.11*numel(Y1Hsd{i}))
    f = fit(T1Hsd{i}(rmi1Hsd(i):rmx1Hsd(i)).', Y1Hsd{i}(rmi1Hsd(i):rmx1Hsd(i)),'gauss1')
    shift1Hsd(i) = f.b1*(sw1H/numel(Y1Hsd{i}))
    low1Hsd(i) = 6833-shift(i)
    hi1Hsd(i) = low1Hsd(i)+sw1H
    X1Hsd{i} = linspace (low1Hsd(i), hi1Hsd(i), numel(Y1Hsd{i}))
    X1Hsd{i} = X1Hsd{i}.'
    [X1Hsdn{i}, Y1Hsdn{i}] = msresample(X1Hsd{i},Y1Hsd{i}, 100000,'RANGE',[0 100000],'Uniform', true,'showplot',true)
end
% delete the old Y1Hdsp every time
Y1Hsdsp = transpose(cell2mat(Y1Hsdn.'));
plot(Y1Hsdsp.');
%% [1Hmsd] Show the 1Hmsd NMR spectra as they are represented
% Yr = flip(Ysp.',1)
X1Hsda = linspace (0, 10, 100000);
plot (X1Hsda, Y1Hsdsp.');
xlabel('shift(ppm)');
ylabel('Intensity');
set(gca,'Xdir','reverse');
%% [1Hmsd] Base-line correction, plot one correction and the stackplot
sf = 610;% best 420
pf =0.005;% best 0.034
ws= 2000;% best 1780
wf = @(mz) sf+ pf .*mz;
%define smothing factor 'sf' and pace factor pf
X1Hsdpt = 1:100000
```

```
Y1Hsdblc = msbackadj(X1Hsdpt.',Y1Hsdsp.','StepSize', wf,'WindowSize',ws,'RegressionMethod','spline','ShowPlot',10);
%in case flip Yblcr = flip(Yblc,1)
figure;
plot(X1Hsdpt,Y1Hsdblc);
ylim([-15 50]);
xlabel('shift(ppm)');
ylabel('Intensity');
set(gca,'Xdir','reverse');
minval =min(Y1Hsdblc);
meanmin = mean(minval);
disp(minval);
disp(meanmin);
%% [1Hmsd] Subtract the baseline height (delete previous BL1Hsd and base1Hsd before you run it)
Y1Hsdbc = Y1Hsdblc.'
BL1Hsd = Y1Hsdbc(:, [700:1100 4000:4700 44500:45300 47600:48600 57819:59000 84360:90000 97490:99000]);
base1Hsd = mean(BL1Hsd,2);
Y01Hsd = Y1Hsdbc-base1Hsd;
plot(Y01Hsd.');
%% [1Hsel] Import spectra, organize those in tables
sp1Hsel = table2array(exp1Hsel42inprogress(:,1:43));
% spec1Hdec = str2double(sp1Hdec);
a1Hsel= round(mean(find(abs(sp1Hsel(:,1)-8.5)<0.0005)));
b1Hsel= round(mean(find(abs(sp1Hsel(:,1)-10.5)<0.0005)));
F1Hselimp = sp1Hsel([a1Hsel:b1Hsel],[2:43]);
%% [1Hsel] Store spectra, spectral witdh, datapoints and resample with reference
sw1Hs = 20000
ns=size(F1Hselimp,2);
X1Hs = cell(ns,1);
Y1Hs = cell(ns,1);
T1Hs = cell(ns,1);
X1Hsn = cell(ns,1);
Y1Hsn = cell(ns,1);
rmi1Hs = zeros(ns,1);
rmx1Hs = zeros(ns,1);
shift1Hs = zeros(ns,1);
low1Hs = zeros(ns,1);
hi1Hs = zeros(ns,1);
for i = 1: ns
    Y1Hs{i} = F1Hselimp(:,i)
    % stse = strfind(Y{i}',[0 0 0])
    %torem = unique([stse, stse+1, stse+2])
    %Y{i}(torem,:) = []
    %Y{i} = flip(Y{i})
    T1Hs{i}= linspace(1,numel(Y1Hs{i}),numel(Y1Hs{i}))
    rmi1Hs(i) = round(0.558*numel(Y1Hs{i}))
    rmx1Hs (i) = round(0.588*numel(Y1Hs{i}))
    f1Hs = fit(T1Hs{i}(rmi1Hs(i):rmx1Hs(i)).', Y1Hs{i}(rmi1Hs(i):rmx1Hs(i)),'gauss2')
    shift1Hs(i) = 0.5*(f1Hs.b1+f1Hs.b2)*(sw1Hs/numel(Y1Hs{i}))
```

```
        low1Hs(i) = 96350-shift1Hs(i)
        hi1Hs(i) = low1Hs(i)+sw1Hs
        X1Hs{i} = linspace (low1Hs(i), hi1Hs(i), numel(Y1Hs{i}))
        X1Hs{i} = X1Hs{i}.'
        [X1Hsn{i}, Y1Hsn{i}] = msresample(X1Hs{i},Y1Hs{i}, 20000,'RANGE',[85000
105000],'Uniform', true,'showplot',true)
    end
    figure;
    Y1Hssp = transpose(cell2mat(Y1Hsn.'));
    plot(Y1Hssp.');
    %% [1Hsel] Show the frequency calibrated NMR spectra as they are represented
    % Y1Hssp(20,:) = Y1Hssp(20,:)/10000;
    X1Hsabs = linspace (8.5, 10.5, 20000);
    plot (X1Hsabs, Y1Hssp.');
    xlabel('shift(ppm)');
    ylabel('Intensity');
    set(gca,'Xdir','reverse');
    %% [1Hsel] Base-line correction, plot one correction and the stackplot
    sf = 400
    pf = 0.008
    ws = 1000
    wf = @(mz) sf+ pf .*mz;
    %define smothing factor 'sf' and pace factor pf
    X1Hspt = 1:20000
    Y1Hsblc = msbackadj(X1Hspt.',Y1Hssp.','StepSize', wf,'WindowSize',ws,'Regression-
Method','spline','ShowPlot',1);
    % define ws =2800
    figure;
    plot(Y1Hsblc);
    ylim([-1e-2 1e-2]);
    %% [1Hsel] Subtract the baseline height
    %Y1Hsbc = Y1Hsblc.'
    %for i = 1:ns
    %     BL1Hs(i,:) = Y1Hsbc(i, [4000:11000 19700:19800])
    %     base1Hs(i) = mean(BL1Hs(i,:))
    %     Y01Hs(i,:) = Y1Hsbc(i,:) - base1Hs(i)
    %end
    %% Other method
    Y1Hsbc = Y1Hsblc.'
    BL1Hs = Y1Hsbc(:, [4000:11000 19700:19800]);
    base1Hs = mean(BL1Hs,2);
    Y01Hs = Y1Hsbc-base1Hs;
    plot(Y01Hs.');
    %% Combination of spectra 1Hd 1Hmsd and 1Hsel
    SPEC1Hd = Y01Hd.';
    SPEC1Hsd = Y01Hsd.';
    SPEC1Hsex = Y01Hs.';
    %phenolref1Hsd = sum(SPEC1Hsd([92000:93000 94700:95500 95900:97000 97300
97900],:));
    %phenolref1Hsex = sum(SPEC1Hsex([7000:8000 9700:10500 10900:12000 12300
12900],:));
```

```
PSPEC1Hsd = max(0, SPEC1Hsd);
PSPEC1Hsex = max(0, SPEC1Hsex);
phenolref1Hsd = sum(PSPEC1Hsd([91000:98000],:));
phenolref1Hsex = sum(PSPEC1Hsex([6000:13000],:));
%% Scale 1Hd and 1Hsel to 1Hmsd
for i = 1:size(SPEC1Hsd,2)
    SPEC1Hdscal(:,i) = SPEC1Hd(:,i)*sum(SPEC1Hsd(27400:28500,i))/sum(SPEC1Hd(27400:28500,i))
    SPEC1Hsexscal(:,i) = SPEC1Hsex(:,i)*phenolref1Hsd(1,i)/phenolref1Hsex(1,i)
end
%% transplant
RICC1H = SPEC1Hsd
RICC1H([8000:9457 1200:14320 15500:16650 19400:21400 22670:24000 41000:42050 42572:43520 53000:54400],:) = SPEC1Hdscal([8000:9457 1200:14320 15500:16650 19400:21400 22670:24000 41000:42050 42572:43520 53000:54400], :);
RICC1H([90000:100000], :) = SPEC1Hsexscal([5000:15000], :);
plot(RICC1H);
%% Normalize the second time spectra and show the level AUC
RICC1Hn = msnorm(X1Hdpt.',RICC1H,'Limits',[22500 24000],'Max',1000)
plot(RICC1Hn);
%% Check reference spectrum (with wanted integral range) before allignment
%Yn1Hd = Y1Hdn.';
RICC1Hh = RICC1Hn.'
%Yn1Hd_ref = Yn1Hd(10,:);failing in this moment
%plot(Yn1Hd_ref);
%ylim([-1.5 100]);
%% Icoshift allignment spectra must be along rows (h)
[RICCal1H, int1H, ind1H] = icoshift('median', RICC1Hh, evoo1Hd_allrng,'b',[2 1 1], X1Hda);
figure;
plot(X1Hda, RICCal1H);
RICC1Hfin = RICCal1H.';
%% Plot output
plot(RICC1Hfin);
xlabel('shift(ppm)');
ylabel('Intensity');
set(gca,'Xdir','reverse');
% figure;
% plot(RICfin_1Hd(:,[1 8 9 21 22 23 25])); %print selected spectra
% xlabel('shift(ppm)');
% ylabel('Intensity');
% set(gca,'Xdir','reverse');
%% intgrng1Hdpt = Intgrng1Hdppm*10000
for i = 1:numel(Intgrng1Hdpt(:,1))
    IntgTbl1H(i,:) = trapz(RICC1Hfin(Intgrng1Hdpt(i,2):Intgrng1Hdpt(i,1),:),1)
end
% normalize tables
for i = 1:ns
    Intab1H(:,i) = IntgTbl1H(:, i)*1000/IntgTbl1H(83,i)
end
% positive integrations
```

```
InTbl1H = max(Intab1H,0);
```

For ^{13}C

```
%% Import data (better normalized data)
spect13C = table2array(exp13C42inprogr);
%F13Cimport = F13C12spettri([73:261554],[2:13])
a13C= round(mean(find(abs(spect13C(:,1)+2)<0.0005)));
b13C= round(mean(find(abs(spect13C(:,1)-198)<0.0005)));
F13Cimport = spect13C([a13C:b13C],[2:43]);
%% Normalize spectra and show the level AUC
X13Corigpt = 1:size(F13Cimport,1);
% spectra running along columns and data-points transponed as well
F13Cn = msnorm(X13Corigpt.',F13Cimport,'Limits',[40000 41500],'Max',1000)
plot(F13Cn);
%% Store spectra, spectral witdh, datapoints and resample with reference
ns=size(F13Cn, 2);
X13C = cell(ns,1);
Y13C = cell(ns,1);
T13C = cell(ns,1);
X13Cn = cell(ns,1);
Y13Cn = cell(ns,1);
rmi13C = zeros(ns,1);
rmx13C = zeros(ns,1);
shift13C = zeros(ns,1);
lima13C = zeros(ns,1);
limb13C = zeros(ns,1);
sw13C = 200000;
for i = 1: ns
    Y13C{i} = F13Cn(:,i)
    %stse = strfind(Y{i}',[0 0 0])
    %torem = unique([stse, stse+1, stse+2])
    %Y{i}(torem,:) = []
    % Y{i} = flip(Y{i})
    % T{i} = flip(T{i})
    T13C{i}= linspace(1,numel(Y13C{i}),numel(Y13C{i}))
    rmi13C(i) = round(0.352*numel(Y13C{i}))
    rmx13C (i) = round(0.364*numel(Y13C{i}))
    f = fit(T13C{i}(rmi13C(i):rmx13C(i)).', Y13C{i}(rmi13C(i):rmx13C(i)),'gauss1')
    shift13C(i) = f.b1*(sw13C/numel(Y13C{i}))
    low13C(i) = 68934 - shift13C(i)
    hi13C(i) = low13C(i)+ sw13C
    X13C{i} = linspace (low13C(i), hi13C(i), numel(Y13C{i}))
    X13C{i} = X13C{i}.'
   [X13Cn{i}, Y13Cn{i}] = msresample(X13C{i},Y13C{i}, 195000,'RANGE',[0 195000],'Uniform', true,'showplot',true)
end
Y13Csp = transpose(cell2mat(Y13Cn.'));
figure;
plot(Y13Csp.');
%% Show the alligned NMR spectra as they are represented
X13Cabs = linspace (0, 195, 195000);
```

```
plot (X13Cabs, Y13Csp);
xlabel('shift(ppm)');
ylabel('Intensity');
set(gca,'Xdir','reverse');
%% Base-line correction, plot one correction and the stackplot
sfC = 900;
pfC =0.01;
wsC= 2800;
wfC = @(mz)sfC+ pfC .*mz;
X13Cpt = 1:195000
Y13Cbc = msbackadj(X13Cpt.',Y13Csp.','StepSize', wfC,'WindowSize',wsC,'RegressionMethod','spline','ShowPlot',2);
figure;
plot(Y13Cbc);
set(gca,'Xdir','reverse');
ylim([-2 10]);
%% Baseline flatting
% transform spectra along columns into spectra along raws
Ybc13C = Y13Cbc.'
BL13C = Ybc13C(:, [12:6000 41000:60000 80000:110000 145000:160000 181000:194000])
base13C = mean(BL13C,2);
Y13C0 = Ybc13C-base13C;
Y013C = Y13C0.';
plot(Y013C);
% for i = 1:ns
% BL(i,:) = Ybc(i, [20:12500 35000:55000 82000:110000 145000:160000 180000:194500])
% base(i) = mean(BL(i,:))
% Y0(i,:) = Ybc(i,:) - base(i)
% end
%% Normalize spectra and show the level AUC
% spectra on different rows and along columns
Y013Cn = msnorm(X13Cpt.',Y013C,'Limits',[29000 30000],'Max',1000)
plot(Y013Cn);
%Yn = normalize(Y0t, 1, "norm",Inf);
%Ynorm = cell2mat(A)
%plot(Ynorm.');
%set(gca,'Xdir','reverse');
%ylim([-0.3 1]);
%% allignment spectra' instead of 'median'
[Y13Cal, int13C1, ind13C1] = icoshift('median', Y013Cn.', evoo13C_all,'b',[2 1 1], X13Cabs);
plot(X13Cpt, Y13Cal);
figure;
Y13Cfin = Y13Cal.';
plot(Y13Cfin);
%% Integrations
for i = 1:size(IntgRng13Cpt,1)
    InTab13C(i,:) = trapz(Y13Cfin(IntgRng13Cpt(i,2):IntgRng13Cpt(i,1),:),1)
end
for i = 1:ns
```

```
    InTbl13C(:,i) = InTab13C(:, i)*100/(3*InTab13C(30,i))
end
InTbl13C = max(InTbl13C,0);
```

S2.2 NMR Data and assignments

Table S1. [1]H targeted integration assignments at the best of our actual extent.

Chemical identification	Integral range	
Saturated linear aldehydes'	9.77	9.74
'5S,4R(TY-EA-DA+HTY-EA-DA)'	9.69	9.66
'HTY-EDA-DA (Oleocanthal 3-C-H)'	9.651	9.6346
'TY-EDA-DA (Olacein 3-C-H)'	9.6345	9.62
b unsaturated aldehyde (A)	9.61	9.557
'HTY-EA-CYA'	9.53	9.519
'TY-EA-CYA+1/2(2E-hexenal)+1/2(2E-alkenal)'	9.5189	9.5036
'1/2(2E-hexenal)'	9.5035	9.495
'1/2(2E-alkenal)'	9.4949	9.49
'5S,4S(TY-EA-DA+HTY-EA-DA)'	9.4899	9.45
'Aldehyde B'	9.37	9.34
'Aldehyde C'	9.33	9.3
'Aldehyde D'	9.2999	9.28
'ELENOLIDE see magiatis et al 2019'	9.2799	9.26
'TY-EDA-DA (Olacein 1-C-H)'	9.244	9.2226
'HTY-EDA-DA (Oleocanthal 1-C-H)+TY-EA-DA+TY-EA-EN'	9.2225	9.21
'HTY-EA-DA+HTY-EA-EN'	9.2099	9.196
'Unidentified water exchanging protons'	8.302	8.106
'unknown singlet'	8.106	8.09
'unidentified hydroxyl groups'	8.09	8.007
'formaldehyde'	8.007	7.996
'phtalate A'	7.728	7.694
'Polyphenol A'	7.683	7.66
'ELENOLIDE-3-CH'	7.65	7.64
'unknown'	7.594	7.58
'TY-EA-CYH (3-CH)'	7.58	7.562
'HTY-EA-CYH (3-CH)'	7.562	7.547
'phtalate B'	7.538	7.502
'CDCl3 sat A'	7.5	7.47

'half 1 TY-EA-CYA'	7.413	7.404
'half 1 HTY-EA-CYA'	7.404	7.397
'half 2 TY-EA-CYA'	7.386	7.378
'half 2 HTY-EA-CYA'	7.378	7.371
'unknown cycloene- eter A'	7.354	7.347
'unknown cycloene - eter B'	7.347	7.338
'CDCl3'	7.302	7.226
'TY-Derivates (4-CH, 2H)'	7.1	7
low intensity polyphenol range	6.9966	6.814
TY+HTY-derivatives (7-CH, 2H & 7,8-CH, 2H)	6.8088	6.6845
maybe 12-HPDOE 12-CH	6.6618	6.6001
11-CH 9-HPDOE + ELENOLIDE-8-CH + 1H del 4-CH HTY	6.6	6.52
unidentified peroxide + maybe phe-nols	6.3036	6.2031
maybe12-HPDOE 10-CH overlapped	6.1296	6.0329
12-CH (Z,E) 9-HPDOE'	6.032	5.96
unknown very small waves	5.9599	5.811
sestuplet -phenols?	5.802	5.692
'(E,E) peroxide 12-HDPOE'	5.6919	5.612
10-CH (Z,E) 9-HPODE'	5.611	5.5356
small interrange	5.534	5.509
13 CH - 9-HPODE	5.5083	5.44
CH=CH	5.4378	5.2903
CH-TG	5.2902	5.223
(8CH)SQ+1H(1,2-DG)	5.19	5.0539
inter range terpenes	5.051	4.8631
very small terpene + exchanging water	4.759	4.7254
overlapped singlet of cycloleuca-lenol+24-MECY+GRAMI+Obtusifo-liol	4.7242	4.695
overlapped singlet of cycloleuca-lenol+24-MECY+GRAMI+Obtusifo-liol	4.674	4.642
Geranylgeraniol and phytol esters (GERG and PHYT)	4.605	4.564
unknown glyceride maybe	4.4	4.3501
CH2''-TG+(1H of 1,2-DG)	4.35	4.2532
1H*(1,2-DG)	4.2525	4.205
CH2'-TG+4*(1,3-DG)	4.2049	4.0998
'1H*(1,3-DG)'	4.0997	4.05

phospholipidic esters maybe	4.0499	4
inter range with very small waves	3.996	3.8495
seldom little double hump	3.8495	3.768
'2H*1,2-DG'	3.76	3.68
tetracosanol and other alchools + H 1BSIT+citrostadienol	3.6757	3.5539
1H-VSTR	3.6	3.4765
singlet 3.47	3.4745	3.4574
inter range	3.4499	3.3254
interval with maybe methanol and Cycloartenol+ 24-Methylenecy-cloartanol (3->CH-OH)+Cicloeuca-lenol+ gramisterol (>CH-OH)	3.3231	3.2435
Cycloeucalenol + oleanolic acid + ursolic acid +uvalol	3.2438	3.1675
Obtusifoliol +b-amyrinyn	3.1668	3.1422
gramisterol +Obtusifoliol+ beta-amyrin	3.1422	3.0639
'maslinic acid '	3.0639	2.9697
'nothing (once satB-divinylCH2)'	2.9117	2.8333
'divynilCH2-TUFA'	2.85	2.789
'divynilCH2-DUFA'	2.7889	2.733
'foot of the divynil signal'	2.7329	2.6988
'unknown a'	2.6983	2.5386
'interrange (once satB-a-CH2)'	2.4781	2.37
a-CH2	2.3699	2.24
'nothing (once sat b-vinylCH2+satA-a-CH2)'	2.2399	2.14
'4H*vinylCH2-pufa+20(H) vinyl CH2-SQ'	2.12	1.93
'space (once sat vin CH2)'	1.9299	1.9143
'interspace'	1.9141	1.8335
other res. Water	1.8335	1.686
'1-SQ-CH3-6H*(SQ)'	1.6859	1.66
b-CH2+18H*(SQ)	1.6599	1.53
'inter range CH2-b-CH2'	1.5299	1.43
'CH2-FA'	1.4299	1.19
'foot of the CH2-FA signal'	1.1899	1.045
'other methylic species'	1.0449	1
'Me-W3-FA'	0.998	0.95
'Me-FA'	0.9499	0.8
URSLA + MSLN A + UVERD	0.7999	0.75

3H - VSTR	0.7	0.66
2H -CYST	0.5961	0.546
3H -GRMST + 3H CYAVCA	0.5459	0.53
1H - CYBR + 2H-CYST	0.35	0.315
1H - CYEUC	0.2	0.12
'TMS'	0.02	0.0001

Table S2. ^{13}C targeted integration assignments at the best of our actual extent. Letter codes are those reported in Table 1 whereas the "i" and "e" notations refer to the internal and external position respectively

Assignments with letter code	Integral Range in ppm*1000	
Pext1+Sext1+Vext1	173266	173233
Oext1+Lext1+POext1+Lnext1	173232	173200
Oint1+Lint1+Pint1+Sint1+POint1+Eint1	172850	172780
SQ10(q)	135070	135000
SQ6(q)	134900	134800
Ln16	132000	131900
SQ2(q)	131200	131150
L13+Ln9	130250	130150
Oint10	130050	130026
Oe10+POi10	130025	130006
Le9+POe10	130005	129990
Li9	129989	129960
V12	129959	129920
unknown	129890	129860
V11	129841	129815
Oext9	129750	129707
Oint9+PO9	129706	129660
Ln12	128330	128285
Ln13	128284	128220
Li10	128140	128108
Le10	128107	128080
L12	127950	127900
Lni10	127810	127795
Lne10	127794	127776
Ln15	127170	127120
SQ3	124500	124430
SQ11	124380	124334
SQ7	124333	124300
2-CHO 1.2DG	72166	72124
2-CHO TG	69100	68800

1-CH2O 1.2DG	65200	64950
1-CH2O TG	62250	61900
3-CH2OH 1.2DG	61600	61400
SQ9	39800	39778
SQ5	39777	39750
C2int 1.2DG	34316	34260
(Pint+Sint+Oint+Vint+POint+Lint+Lnint)2	34259	34150
C2e 1.2DG (2C)	34140	34100
Pext2+Sext2+Vext2	34099	34065
Oext2+Lext2+Lnext2	34064	34000
P14+S16	32000	31966
O16	31965	31900
V16	31850	31824
PO14	31823	31800
L16	31590	31550
PO13	29960	29930
O12+V9	29870	29790
V14+S12	29789	29770
S8+S9+S10+S11+S13+S14+O7+P12+P11+P10+PO12= 6S+3P+O+PO	29769	29721
P8+P9+Si7 = 2P+1Si	29720	29690
P7+Se7+Li7= 1P+1Se+Li	29685	29656
Le7	29655	29635
Ln7	29634	29619
	29618	29600
O14+Si5+Ve8	29599	29553
Pi5+Se5+Vi8	29552	29538
Pe5	29537	29505
Ve7+Vi7	29504	29473
	29472	29440
S15	29439	29425
P13	29424	29401
L15	29400	29386
O15+O13+V15+Si6	29385	29340
P6+V5+V6+Se6	29339	29290
Oi5+Li5+Lni5	29265	29228
Oe5+Le5+Lne5+POi5	29227	29190
O6+L6+Ln6+Pe4+S4	29189	29138
Oe4+Pi4+Le4+Lne4	29137	29105
Oi4+Li4+Lni4	29104	29060
V4	29040	29020
SQ12	28320	28300

O11+V13	27300	27247
L14+V10+Ln8+PO11	27246	27233
L8	27232	27221
O8+PO8	27220	27150
SQ4	26830	26780
SQ8	26720	26660
SQ1	25740	25690
L11+Ln11	25689	25620
Ln14	25590	25540
Oi3+Pi3+Li3+Si3	24960	24914
Pe3+Vi3+Se3+Lni3	24913	24896
Oe3+Le3+Ve3+Lne3+PO3	24895	24850
P15+S17	22750	22731
O17	22730	22708
V17	22707	22680
L17	22630	22600
PO15	22599	22560
Ln17	20700	20500
SQ13	17800	17600
SQ14	16070	16034
SQ15	16030	16000
Ln18	14320	14283
unk	14282	14245
O18+V18+P16+S18	14200	14109
L18+PO16	14108	14050
UNK(maybe satellite)	14000	13950
TMS	32	1

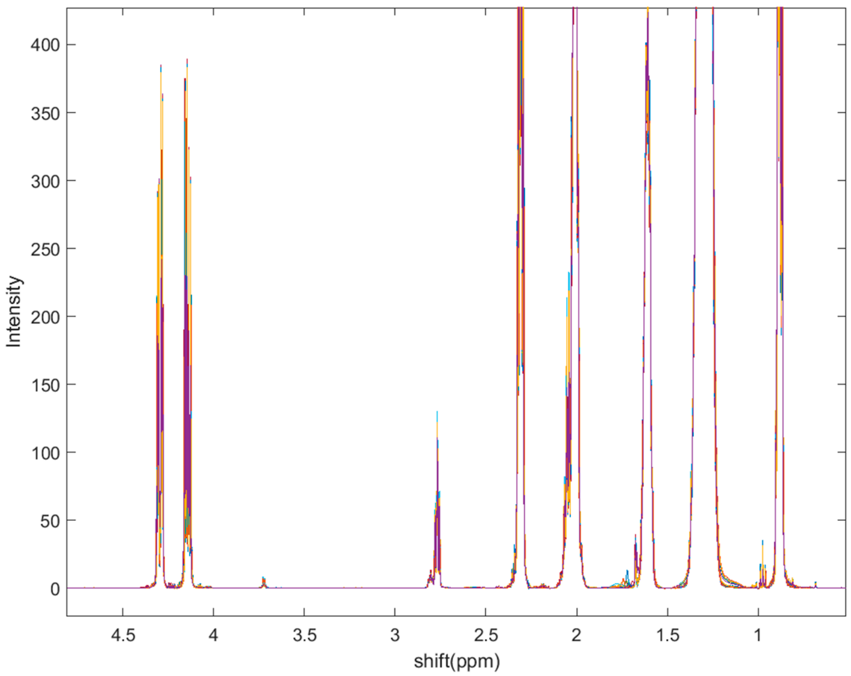

Figure S1. Overlapped ^{1}H aligned traces restored by three experiments (RICC-NMR) for all the analysed samples.

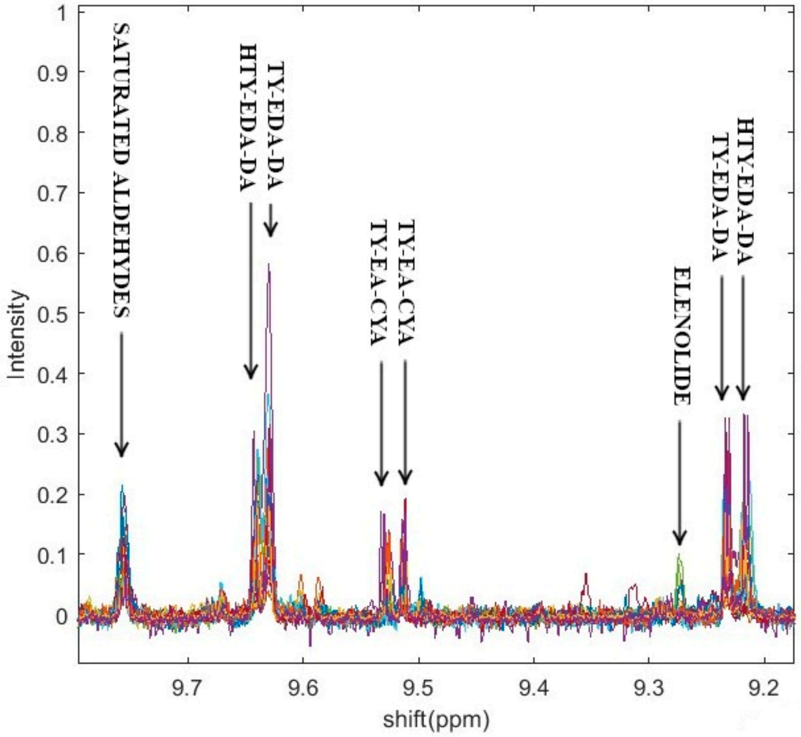

Figure S2. Staking of the ^{1}H aldehydic region aligned for the analysed samples and main assignments determining important metabolites for the quantification.

Data conversion into quantification variables is basically attained by the MARA-NMR algorithm which minimizes the mean-square deviations between theoretical and experimental integrations to fit a specific composition profile [26].

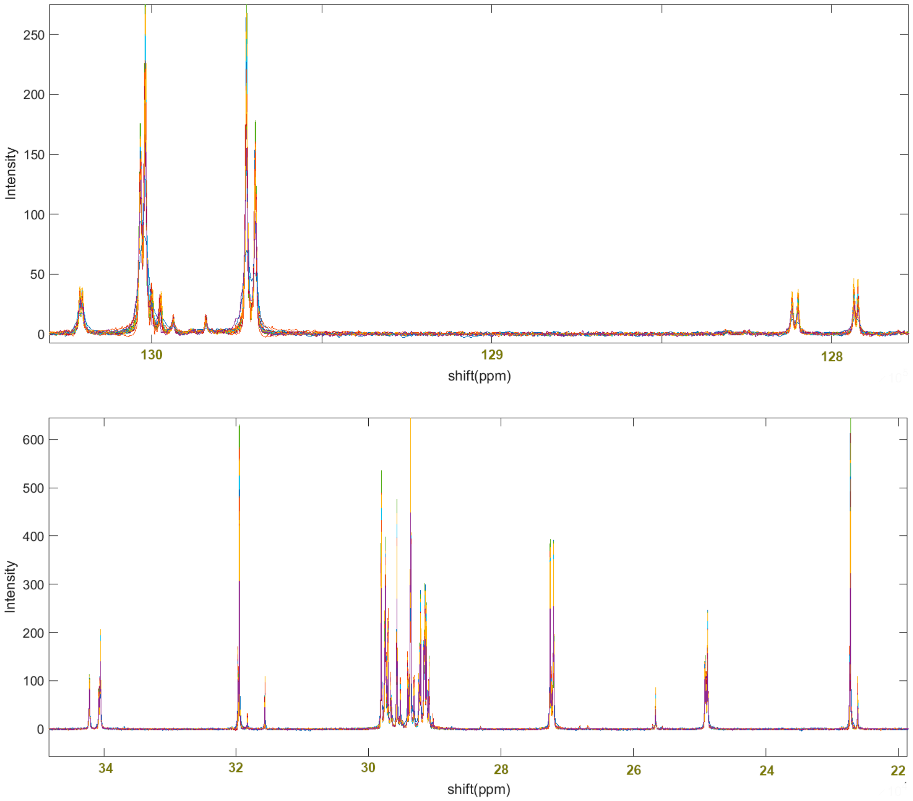

Figure S3. [13]C stack-plot for some selected regions, icoshift alignment allows fair integration and quantification of signals.

The careful assignment of most of the integrations for the ^{1}H and ^{13}C profile reported in Tables S1 and S2 allowed the correct set of specific relationships making up the MARA-NMR method.

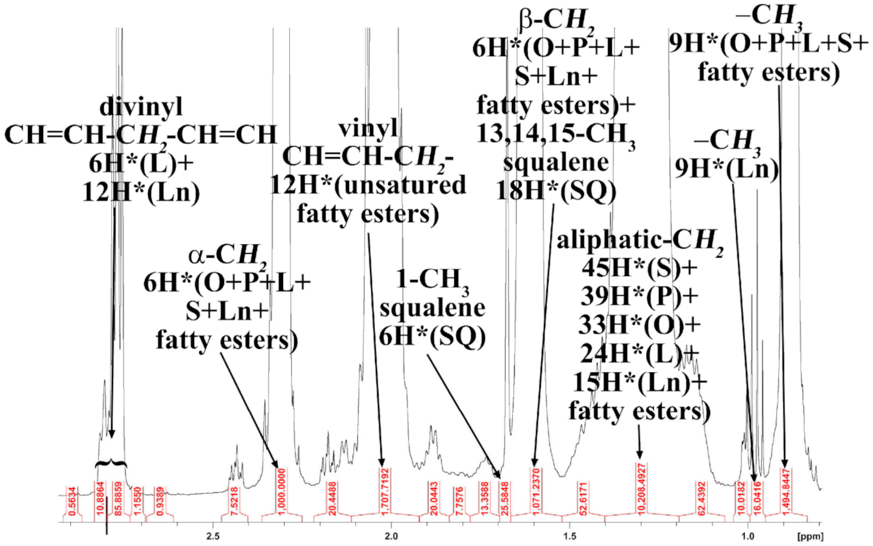

Figure S4. ^{1}H- NMR profile with the assignment of the main signals (letter codes are consistent with Table 1).

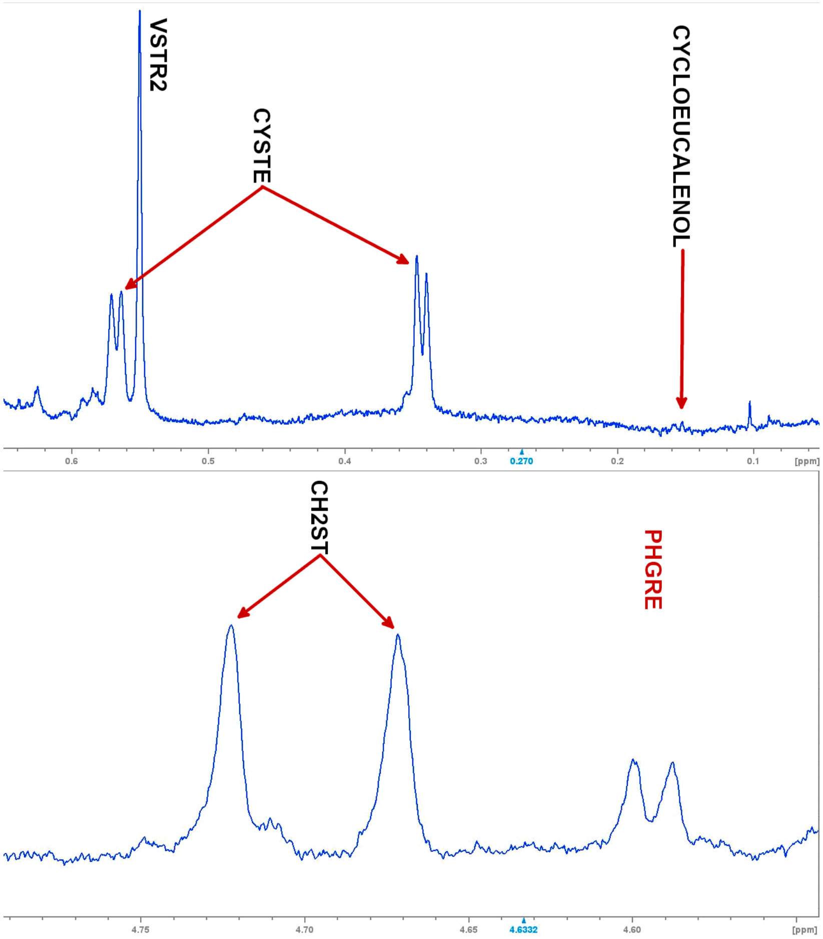

Figure S5. ^{1}H- NMR profile of the multi-suppressed and ^{13}C decoupled spectrum with the assignment of the main signals (letter codes are consistent with Table 1).

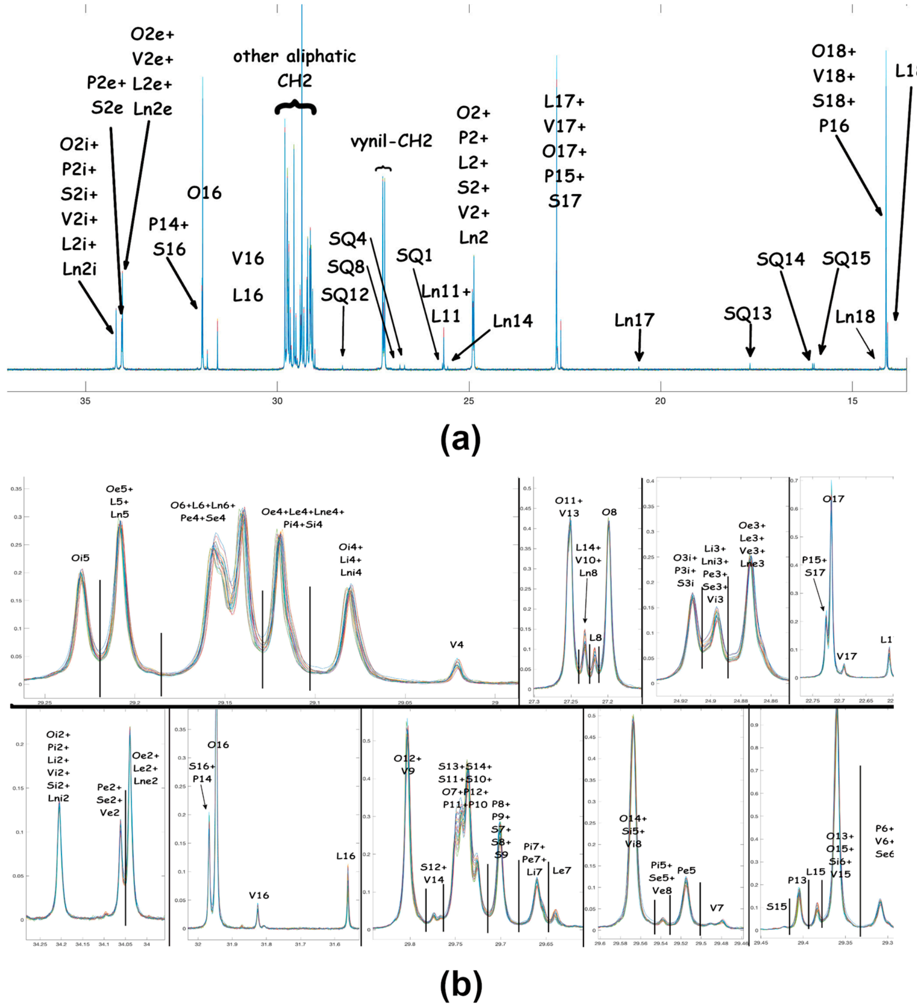

Figure S6. ^{13}C stack-plots with the assignment for fatty acids and squalene (with the letter code reported in Table 1 and the numbers indicating the IUPAC position in the specified molecule), (a) general assignments in the aliphatic region and (b) details of specific narrow windows.

S2.3 Quantified compounds

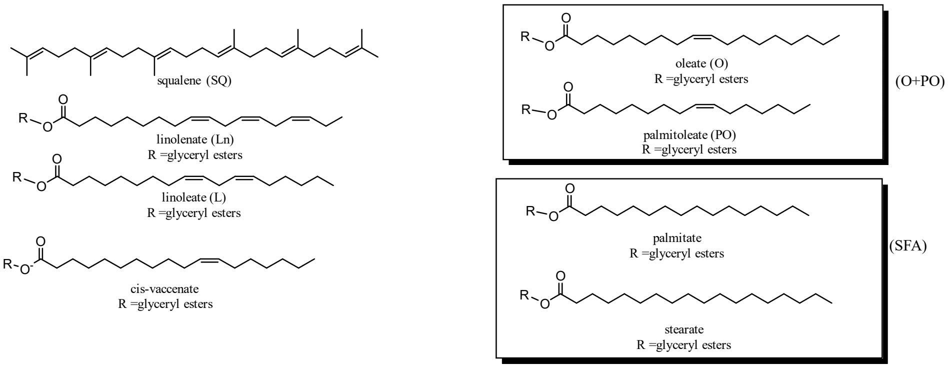

Figure S7. Molecular structure of the quantified fatty esters reported in Table 1 with the relative code. All the codes with the "2" notation in Table 1 refer to the esterification in the 2 (second) position.

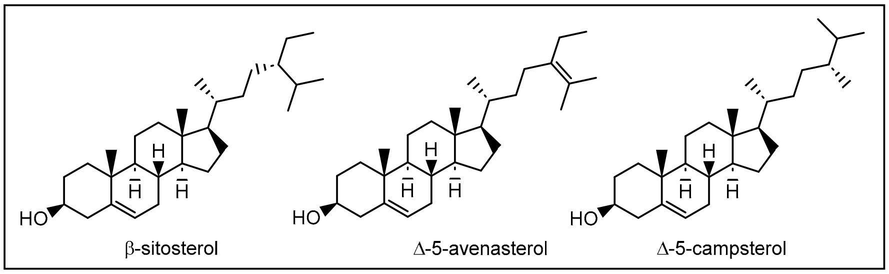

Vegetal Sterols (VSTR)

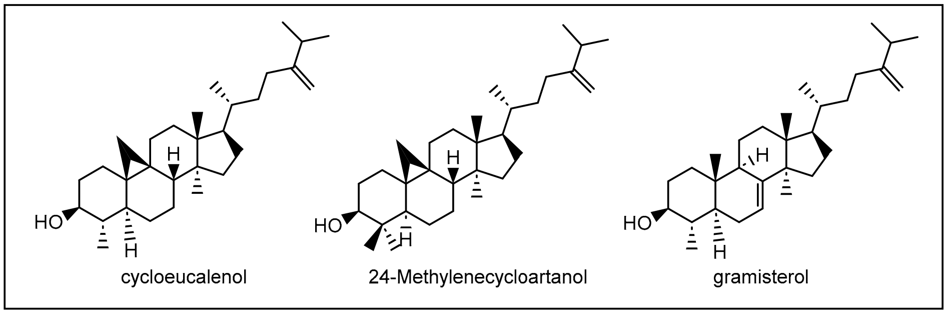

Vegetal sterols with terminal methylene residue (CH2ST)

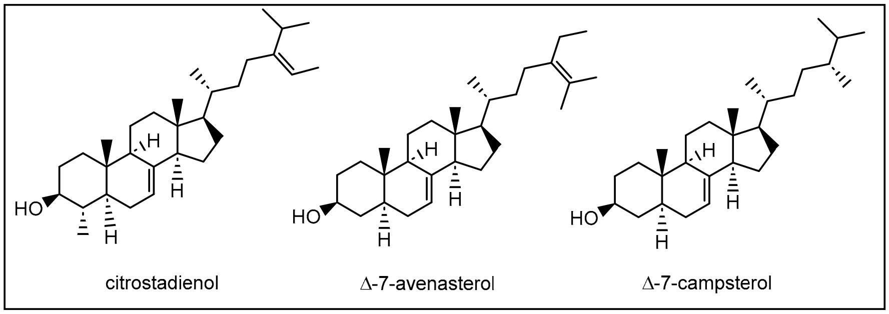

Vegetal Sterols second group (VSTR2)

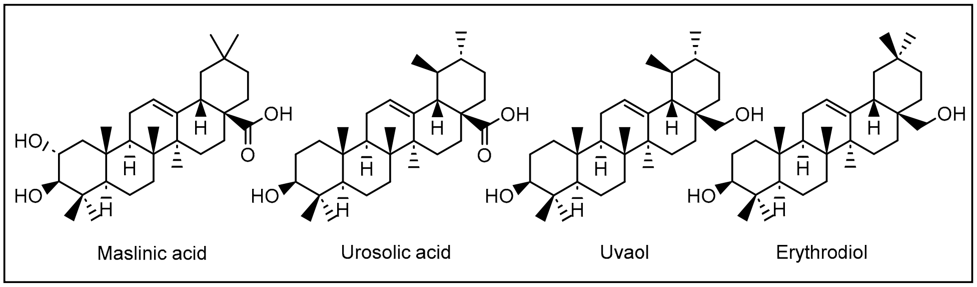

Carboxy and hydroxilated sterols (MUUVER)

Figure S8. Molecular structure of the quantified sterols reported in Table 1 with the relative code.

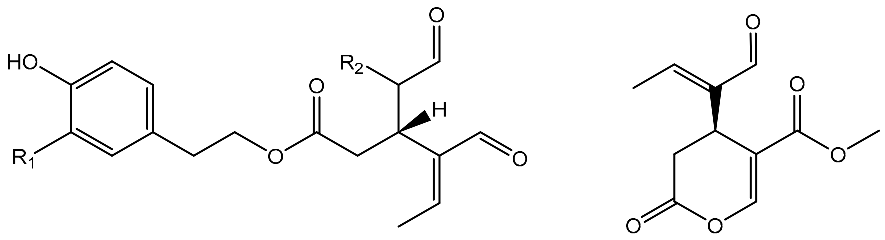

R_1=H;	R_2=H;	Oleocanthal	(TY-EDA)	Elenolide (ELNL)
R_1=OH;	R_2=H;	Oleacein	(HTY-EDA)	
R_1=H;	R_2=COOMe;	Ligstroside aglycone	(TY-EA)	
R_1=OH;	R_2=COOMe;	Oleuropein aglycone	(HTY-EDA)	

Figure S9. Molecular structure of the quantified phenolic species as reported in Table 1 with the relative code.

References

1. Esposto, S.; Taticchi, A.; Urbani, S.; Selvaggini, R.; Veneziani, G.; Di Maio, I.; Sordini, B.; Servili, M. Effect of Light Exposure on the Quality of Extra Virgin Olive Oils According to Their Chemical Composition. *Food Chem.* **2017**, *229*, 726–733.
2. Boskou, D.; Tsimidou, M.; Blekas, G. *Olive Oil: Chemistry and Technology*, 2nd ed.; AOCS Publishing: Champaign, IL, USA, 2015.
3. Salvo, A.; Tuttolomondo, A. The Role of Olive Oil in Cardiometabolic Risk. *Metabolites* **2025**, *15*, 190.
4. Gorzynik-Debicka, M.; Przychodzen, P.; Cappello, F.; Kuban-Jankowska, A.; Gammazza, A.M.; Knap, N.; Wozniak, M.; Gorska-Ponikowska, M. Potential Health Benefits of Olive Oil and Plant Polyphenols. *Int. J. Mol. Sci.* **2018**, *19*, 686.
5. Martín-Peláez, S.; Covas, M.I.; Fitó, M.; Kušar, A.; Pravst, I. Health Effects of Olive Oil Polyphenols: Recent Advances and Possibilities for the Use of Health Claims. *Mol. Nutr. Food Res.* **2013**, *57*, 760–771.
6. Xiang, C.; Xu, Z.; Liu, J.; Li, T.; Yang, Z.; Ding, C. Quality, Composition, and Antioxidant Activity of Virgin Olive Oil from Introduced Varieties at Liangshan. *LWT* **2017**, *78*, 226–234.
7. Kouka, P.; Priftis, A.; Stagos, D.; Angelis, A.; Stathopoulos, P.; Xinos, N.; Skaltsounis, A.L.; Mamoulakis, C.; Tsatsakis, A.M.; Spandidos, D.A.; et al. Assessment of the Antioxidant Activity of an Olive Oil Total Polyphenolic Fraction and Hydroxytyrosol from a Greek Olea Europea Variety in Endothelial Cells and Myoblasts. *Int. J. Mol. Med.* **2017**, *40*, 703–712.
8. Andrewes, P.; Busch, J.L.H.C.; De Joode, T.; Groenewegen, A.; Alexandre, H. Sensory Properties of Virgin Olive Oil Polyphenols: Identification of Deacetoxy-Ligstroside Aglycon as a Key Contributor to Pungency. *J. Agric. Food Chem.* **2003**, *51*, 1415–1420.
9. The European Commission. II of the European Parliament and of the Council as Regards Marketing Standards for Olive Oil, and Repealing Commission Regulation (EEC) No 2568/91 and Commission Implementing Regulation (EU) No 29/2012. *Off. J. Eur. Union* **2022**, *L 284*, 1–22.
10. Bella, G.; Rotondo, A. Theoretical Prediction of ^{13}C NMR Spectrum of Mixed Triglycerides by Mean of GIAO Calculations to Improve Vegetable Oils Analysis. *Chem. Phys. Lipids* **2020**, *232*, 104973.
11. Revelou, P.K.; Pappa, C.; Kakouri, E.; Kanakis, C.D.; Papadopoulos, G.K.; Pappas, C.S.; Tarantilis, P.A. Discrimination of botanical origin of olive oil from selected Greek cultivars by SPME-GC-MS and ATR-FTIR spectroscopy combined with chemometrics. *J. Sci. Food Agric.* **2021**, *101*, 2994–3002.
12. Carvalho, M.S.; Mendonça, M.A.; Pinho, D.M.M.; Resck, I.S.; Suarez, P.A.Z. Chromatographic Analyses of Fatty Acid Methyl Esters by HPLC-UV and GC-FID. *J. Braz. Chem. Soc.* **2012**, *23*, 763–769.
13. Ammar, S.; Kelebek, H.; Zribi, A.; Abichou, M.; Selli, S.; Bouaziz, M. LC-DAD/ESI-MS/MS Characterization of Phenolic Constituents in Tunisian Extra-Virgin Olive Oils: Effect of Olive Leaves Addition on Chemical Composition. *Food Res. Int.* **2017**, *100*, 477–485.
14. Ruiz-Aracama, A.; Goicoechea, E.; Guillén, M.D. Direct Study of Minor Extra-Virgin Olive Oil Components without Any Sample Modification. ^{1}H NMR Multisupression Experiment: A Powerful Tool. *Food Chem.* **2017**, *228*, 301–314.
15. Calò, F.; Girelli, C.R.; Angilè, F.; Del Coco, L.; Mazzi, L.; Barbini, D.; Fanizzi, F.P. ^{1}H-NMR Profiling Shows as Specific Constituents Strongly Affect the International EVOO Blends Characteristics: The Case of the Italian Oil. *Molecules* **2021**, *26*, 2233.

16. Maestrello, V.; Solovyev, P.; Bontempo, L.; Mannina, L.; Camin, F. Nuclear Magnetic Resonance Spectroscopy in Extra Virgin Olive Oil Authentication. *Compr. Rev. Food Sci. Food Saf.* **2022**, *21*, 4056–4075.
17. Simmler, C.; Napolitano, J.G.; McAlpine, J.B.; Chen, S.N.; Pauli, G.F. Universal Quantitative NMR Analysis of Complex Natural Samples. *Curr. Opin. Biotechnol.* **2014**, *25*, 51–59.
18. Cevallos-Cevallos, J.M.; Reyes-De-Corcuera, J.I.; Etxeberria, E.; Danyluk, M.D.; Rodrick, G.E. Metabolomic Analysis in Food Science: A Review. *Trends Food Sci. Technol.* **2009**, *20*, 557–566.
19. Belaj, A.; Ninot, A.; Gómez-Gálvez, F.J.; El Riachy, M.; Gurbuz-Veral, M.; Torres, M.; Lazaj, A.; Klepo, T.; Paz, S.; Ugarte, J.; et al. Utility of EST-SNP Markers for Improving Management and Use of Olive Genetic Resources: A Case Study at the Worldwide Olive Germplasm Bank of Córdoba. *Plants* **2022**, *11*, 921.
20. Rotondo, A.; Bartolomeo, G.; Spanò, I.M.; La Torre, G.L.; Pellicane, G.; Molinu, M.G.; Culeddu, N. Comparison between Traditional and Novel NMR Methods for the Analysis of Sicilian Monovarietal Extra Virgin Olive Oils: Metabolic Profile Is Influenced by Micro-Pedoclimatic Zones. *Molecules* **2024**, *29*, 4532.
21. Klikarová, J.; Rotondo, A.; Cacciola, F.; Česlová, L.; Dugo, P.; Mondello, L.; Rigano, F. The Phenolic Fraction of Italian Extra Virgin Olive Oils: Elucidation Through Combined Liquid Chromatography and NMR Approaches. *Food Anal. Methods* **2019**, *12*, 1759–1770.
22. Angilè, F.; Coco, L.D.; Girelli, C.R.; Calò, F.; Mazzi, L.; Fanizzi, F.P.; Vivaldi, G.A.; Camposeo, S. Proton Nuclear Magnetic Resonance (^{1}H NMR) Metabolic Profiles Discriminate Two Monovarietal Extra Virgin Olive Oils, Cultivars Arbequina and Koroneiki, with Different Geographical Origin. *Horticulturae* **2023**, *9*, 66.
23. Dugo, G.; Rotondo, A.; Mallamace, D.; Cicero, N.; Salvo, A.; Rotondo, E.; Corsaro, C. Enhanced Detection of Aldehydes in Extra-Virgin Olive Oil by Means of Band Selective NMR Spectroscopy. *Phys. A Stat. Mech. Appl.* **2015**, *420*, 258–264.
24. Rastrelli, F.; Schievano, E.; Bagno, A.; Mammi, S. NMR quantification of trace components in complex matrices by band-selective excitation with adiabatic pulses. *Magn. Reson. Chem.* **2009**, *47*, 868–872.
25. Savorani, F.; Tomasi, G.; Engelsen, S.B. icoshift: A versatile tool for the rapid alignment of 1D NMR spectra. *J. Magn. Res.* **2010**, *202*, 190–202.
26. Rotondo, A.; Mannina, L.; Salvo, A. Multiple Assignment Recovered Analysis (MARA) NMR for a Direct Food Labeling: The Case Study of Olive Oils. *Food Anal. Methods* **2019**, *12*, 1238–1245.
27. Trygg, J.; Wold, S. Orthogonal Projections to Latent Structures (O-PLS). *J. Chemom.* **2002**, *16*, 119–128.
28. Eriksson, L.; Trygg, J.; Wold, S. CV-ANOVA for Significance Testing of PLS and OPLS® Models. *J. Chemom.* **2008**, *22*, 594–600.
29. Piravi-Vanak, Z.B.; Ghasemi, J.B.; Ghavami, M.; Ezzatpanah, H.; Zolfonoun, E. The influence of growing region on fatty acids and sterol composition of Iranian olive oils by unsupervised clustering methods. *J. Am. Oil Chem. Soc.* **2012**, *89*, 371–378.
30. Rey-Giménez, R.; Sanchez-Gimeno, A.C. Effect of cultivar and environment on chemical composition and geographical traceability of Spanish olive oil. *J. Am. Oil Chem. Soc.* **2024**, *101*, 371–382.
31. Uncu, O.; Ozen, B. Importance of some minor compounds in olive oil authenticity and quality. *Trends Food Sci. Technol.* **2020**, *100*, 164–176.
32. Willis, C.E.; Perlack, R.D. Multicollinearity: Effects, Symptoms, and Remedies. *J. Northeast. Agric. Econ. Counc.* **1978**, *7*, 55–61.
33. Pianeta PSR. Available online: https://www.pianetapsr.it/flex/cm/pages/ServeBLOB.php/L/IT/IDPagina/3012 (accessed on 15 January 2025).
34. Pierantozzi, P.; Torres, M.; Contreras, C.; Stanzione, V.; Tivani, M.; Gentili, L.; Mastio, V.; Searles, P.; Brizuela, M.; Fernández, F.; et al. Phenolic content and profile of olive fruits: Impact of contrasting thermal regimes in non-Mediterranean growing environments. *Eur. J. Agron.* **2025**, *164*, 127506.
35. Lukić, M.; Lukić, I.; Moslavac, T. Sterols and Triterpene Diols in Virgin Olive Oil: A Comprehensive Review on Their Properties and Significance, with a Special Emphasis on the Influence of Variety and Ripening Degree. *Horticulturae* **2021**, *7*, 493.
36. Baldoni, L.; Cultrera, N.G.; Mariotti, R.; Ricciolini, C.; Arcioni, S.; Vendramin, G.G.; Buonamici, A.; Porceddu, A.; Sarri, V.; Ojeda, M.A.; et al. A consensus list of microsatellite markers for olive genotyping. *Mol. Breed.* **2009**, *24*, 213–231.
37. Marchese, A.; Bonanno, F.; Marra, F.P.; Trippa, D.A.; Zelasco, S.; Rizzo, S.; Giovino, A.; Imperiale, V.; Ioppolo, A.; Sala, G.; et al. Recovery and genotyping ancient Sicilian monumental olive trees. *Front. Conserv. Sci.* **2023**, *4*, 1206832
